# Extensive Contamination of Water with Saxitoxin Near the Dam of the Irkutsk Hydropower Station Reservoir (East Siberia, Russia)

**DOI:** 10.3390/toxins10100402

**Published:** 2018-10-01

**Authors:** Mikhail Grachev, Ilya Zubkov, Irina Tikhonova, Maria Ivacheva, Anton Kuzmin, Elena Sukhanova, Ekaterina Sorokovikova, Galina Fedorova, Aleksandr Galkin, Maria Suslova, Olga Netsvetayeva, Elena Eletskaya, Tatyana Pogadaeva, Vladimir Smirnov, Andrey Ivanov, Vladimir Shagun, Viktor Minaev, Olga Belykh

**Affiliations:** 1 Limnological Institute, Siberian Division of the Russian Academy of Science, 3 Ulan-Batorskaya str., 664033 Irkutsk, Russia; grachev@lin.irk.ru (M.G.); ilyagosldstein@gmail.com (I.Z.); iren@lin.irk.ru (I.T.); ivacheva@gmail.com (M.I.); sukhanova@lin.irk.ru (E.S.); kati_sor@mail.ru (E.S.); fedorova@lin.irk.ru (G.F.); suslova@lin.irk.ru (M.S.); r431@lin.irk.ru (O.N.); Lilitanna@lin.irk.ru (E.E.); tatyana@lin.irk.ru (T.P.); minaev@lin.irk.ru (V.M.); belykh@lin.irk.ru (O.B.); 2 A.E. Favorsky Irkutsk Institute of Chemistry, Siberian Division of the Russian Academy of Science, 1 Favorsky str., 664033 Irkutsk, Russia; smirnov@irioch.irk.ru (V.S.); ivanov@irioch.irk.ru (A.I.); shagun@irioch.irk.ru (V.S.); 3 Stylab Company, 5 Zvenigorodskoye hwy., 123022 Moscow, Russia; info@stylab.ru (A.G.)

**Keywords:** cyanobacteria, *Dolichospermum lemmermannii*, saxitoxin, Lake Baikal, Irkutsk reservoir, HPLC-MS, ELISA, hydrochemical analysis

## Abstract

An area of discolored water 50 m wide and 30 m long was found in September 2017 close to the dam of the Irkutsk hydroelectric power station. Water from this spot was sampled for investigation in the present study. Microscopic analysis revealed that the suspended matter in the sample was composed of clumps of filaments, vegetative cells, akinetes and heterocysts that formed short filaments and solitary cells. This matter was found to consist of partially degraded cells of the cyanobacterium *Dolichospermum lemmermannii*. Nucleotide sequencing of DNA isolated from the biomass revealed the presence of the *sxtA* gene which is involved in the synthesis of saxitoxin. Water from the polluted area contained 600 ± 100 μg L^−1^ saxitoxin as measured by HPLC-MS with pre-column modification of the toxin with 2,4-dinitrophenylhydrazine. Immunoassay analysis (ELISA) showed a concentration of saxitoxins in the water of 2900 ± 900 μg L^−1^. Hydrochemical and microbiological analyses suggested the contaminated area appeared as a result of a *D. lemmermannii* bloom, followed by its decay and release of saxitoxin and nutrients. The present paper describes the results of a case study. Better understanding of the phenomenon will depend on the possibility to perform implementation of a large-scale monitoring program.

## 1. Introduction

**Figure 1:**
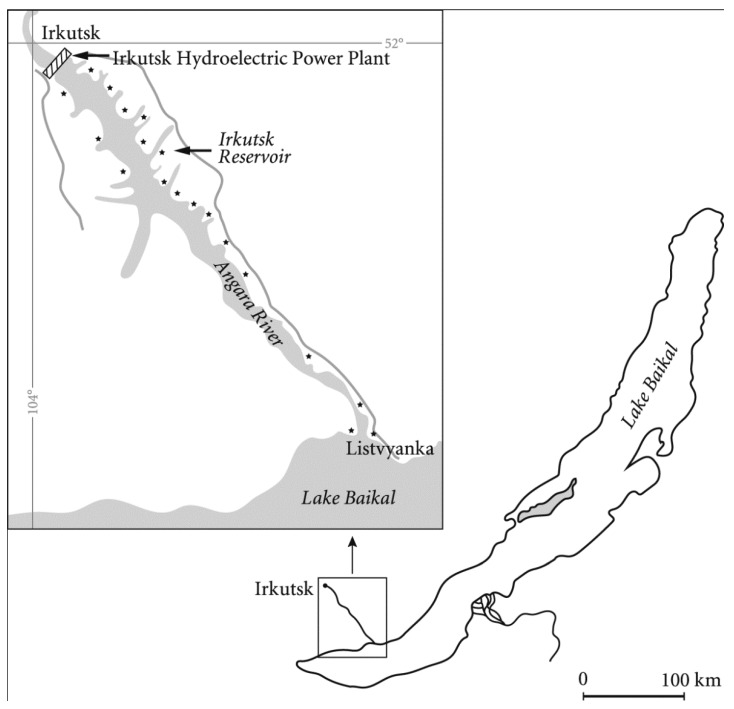
Irkutsk Reservoir and its location relative to Lake Baikal.

The dam of the Irkutsk hydroelectric power station is located 56 km away from the outlet of the Angara River, which originates in Lake Baikal (Figure 1). Lake Baikal is the largest lake in the world, and stores 23 000 km^3^ of fresh water, accounting for 90% of the surface fresh water of Russia. The Irkutsk Reservoir—formed by the dam of the Irkutsk hydroelectric power station—has a volume of 2.1 km^3^, a mean depth of 13.6 m and a maximum depth of 35 m [1]. The residence time of water in the reservoir is about 13 days. Water for turbines is taken at a depth of 25 m, which results in floating substances accumulation on the surface of the water near the dam. Many private households, hotels and recreational buildings, as well as part of Irkutsk city, are situated on the shores of the reservoir.

In late August 2017, the power station administration noticed a discolored and malodorous patch approximately 50 m long and 30 m wide on the surface of water leaning against the dam. The administration wondered whether polluted water from this patch could penetrate drinking water supply facilities. At the request of the administration, we took samples from this patch on 1 September 2017 in order to determine its nature and origin. Preliminary inspection revealed the presence of decaying cells of cyanobacterium *Dolichospermum lemmermannii. D. lemmermannii*, at that time called *Anabaena* sp. [2,3,4], was long ago detected in the plankton of the shallow waters of Lake Baikal [5,6,7,8]. In some years *D. lemmermannii* has been found at very high concentrations—up to 10 × 10^6^ cells L^−1^ [8,9]. During intensive blooms, *D. lemmermannii* can penetrate the surface waters of open Lake Baikal, forming ribbons a few kilometers long. *D. lemmermannii* found in freshwater can produce a neuroparalytic toxin—saxitoxin and its analogues. This hazardous toxin was found recently in the biomass of *D. lemmermannii* inhabiting Lake Baikal, as well as in the water of the lake, but its concentration semi-quantitatively determined by immunoassay analysis (ELISA) was small [10,11], mostly under 3 μg L^−1^, the value which is a health alert level for drinking water in a few countries [12]. To perform quantitative analysis, we developed a new method for saxitoxin determination in water based on HPLC-MS with precolumn modification of saxitoxin with 2,4-dinitrophenylhydrazine.

Saxitoxin and its analogues, often referred to paralytic shellfish toxins (PST), are produced by dinoflagellates in seas and function as sodium channels blockers. They are especially dangerous for humans during the red tides caused by dinoflagellates blooms. They are consumed and accumulated at high concentrations by sea organisms, especially shellfish. Shellfish tissues are often consumed by humans as seafood, and in this way saxitoxin and its analogues penetrate the human body. The mechanism of their neuroparalytic activity is absolutely different from that of synthetic poisons attacking cholinesterase, and the symptoms of intoxication are also dramatically different. In cases of severe intoxication, paralytic shellfish poisoning (PSP) leads to respiratory paralysis and death [13]. On a global basis, almost 2000 cases of human PSP are reported per year, with a 15% mortality rate [14]. The patient can die if there is no proper treatment, including artificial pulmonary ventilation. If medical care is provided, patients usually completely recover. As for freshwater bodies, saxitoxin and its analogues in them are produced by cyanobacteria. Saxitoxin and its analogues produced by cyanobacteria were formerly found in eutrophic and hot environments, but during the last decade, they have also been found in many freshwater bodies of different trophic status worldwide, including temperate and arctic climatic zones [15,16,17]. Safety regulations for saxitoxin and its analogues in freshwater bodies have been introduced in a few countries [12], and many governments have already agreed to implement monitoring of saxitoxins in freshwater, since they evidently create a risk for human health [12].

The present paper describes the results of a case study of the discolored water patch found leaning against the dam of the Irkutsk hydroelectric power plant in the late August 2017. We concentrated our efforts on the detection and determination of saxitoxin since *D. lemmermannii* dominated in the sample from the patch, and because other cyanotoxins have not been detected in its biomass, at least in Lake Baikal, and because saxitoxin is very dangerous, with an acute toxicity (LD_50_ for mice is 263 μg kg^−1^, orally, LD_50_ for adult men is 5.7 μg kg^−1^, orally [18]), and is listed in Schedule 1 of the Chemical Weapons Convention, placing it alongside mustard gases, sarin, ricin and etc. [19]. Consideration of other cyanotoxins was thus beyond the scope of our paper.

## 2. Results

The sample taken near the dam of the Irkutsk reservoir contained a large amount of detritus. The color varied from milk-white to green and the water was malodorous.

### 2.1. Morphology

**Figure 2:**
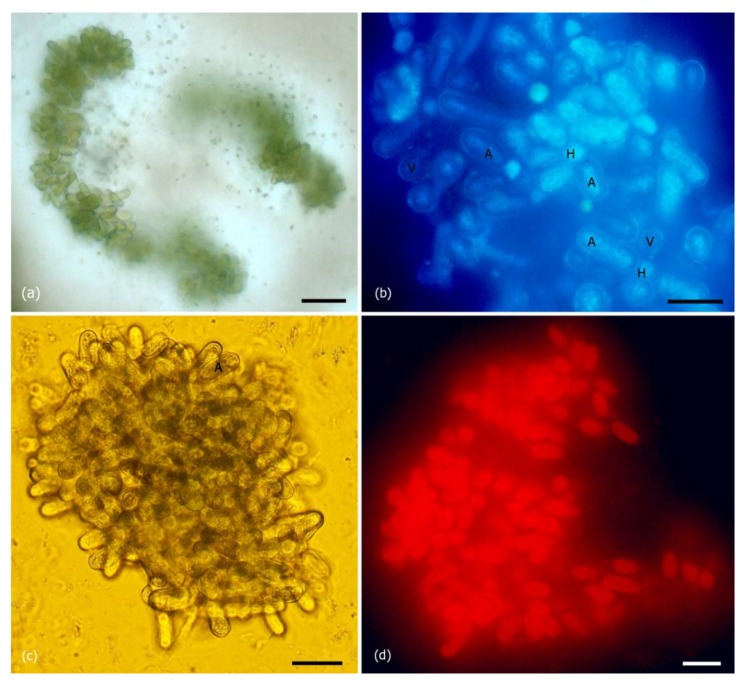
General view of *Dolichospermum lemmermannii* in the sample of water from the Irkutsk Reservoir: (**a**,**с**) light microscopy, (**b**,**d**) epifluorescence microscopy: (**b**) DAPI dye, (**d**) autofluorescence of phicobilins in akinetes. Notations: А—akinetes, Н—heterocysts, V—vegetative cells. Scale: (**а**)—50 μm, (**b**–**d**)—20 μm.

Microscopic examination revealed the presence of microbial filaments mostly in free-floating clumps, rare solitary filaments and separate cells including vegetative cells, heterocysts and akinetes (Figure 2). Clumps were at different stages of degradation, from almost intact to solitary dispersed filaments. Vegetative cells in filaments were almost completely lysed. Remaining vegetative cells were slightly elongated, 6.0 μm in diameter (SD—0.68 μm) and 8.4 μm long (SD—1.1 μm). Cylindrical akinetes (dormant cells with thick walls) were the most numerous cells identified. They were 10.9 μm in diameter (SD—1.08 μm) and 22.0 μm long (SD—2.8 μm). Heterocysts were almost spherical, approximately 7.2 μm in diameter (SD—0.6 μm). 4′-6-Diamidino-2-phenylindole (DAPI) staining demonstrated the accumulation of nucleic acids in akinetes, their homogeneous dispersion over the entire akinetes volume. Fluorescence microscopy revealed intact vegetative cells and akinetes emitted bright orange light under excitation at 546 nm due to the presence of the auxiliary photosynthetic pigments (phycobilins).

Morphological criteria such as width and length of the vegetative cells, akinetes and heterocysts, as well as location of akinetes and heterocysts in trichomes, suggests this species to be cyanobacterium *Dolichospermum lemmermannii* (Richter) P.Wacklin, L.Hoffmann & J.Komárek belonging to the family Aphanizomenonaceae, order Nostocales [3,4], formerly named *Anabaena lemmermannii* [2].

### 2.2. Genetic Analysis

*Dolichospermum* cyanobacteria (*D. circinale* [*Anabaena circinalis*], *D. flos-aquae and D. planctonicum*) are known to synthesize saxitoxins using modular multienzyme complexes composed of polyketide synthase, several other enzymes and carrier proteins encoded by the *sxtA-Z* gene cluster [20,21]. Therefore, we tested the cyanobacterial biomass from the polluted water sample for the *sxtA* gene involved in the synthesis of saxitoxin and its analogues.

The results of PCR with primers designed for amplification of the *sxt*A gene confirmed its presence. The amplification products were cloned and 10 clones were sequenced. These clones were 100% identical to each other. The 555-nucleotide-long sequence showed 99–100% sequence identity to *sxt*A genes of the *Aphanizomenon*, *Anabaena* and *Dolichospermum* genera. The nucleotide sequence of the *sxt*A gene from biomass from the polluted water sample belongs to the cluster of *sxtA* genes from genera of the Nostocales order. The GenBank database contains only a few *sxt*A gene sequences from the *Dolichospermum* genus (namely, genes from *D. planctonicum*, *D. circinale*, *D. flos-aquae*). These species are grouped into a separate cluster on the phylogenetic tree with *Anabaenopsis elenkinii* and species of the *Aphanizomenon* genus. The phylogenetic relationships between representatives of genera of the Nostocales order have not yet been determined, so neither generic nor specific identification down to the species level was possible. If the *sxtA* gene were absent, it would mean that the cyanobacteria present in the polluted water do not produce saxitoxin and its analogues. The presence of this gene suggested that the species studied could potentially produce saxitoxins. Therefore, our next step was to detect saxitoxin in the biomass and in water of the sample.

### 2.3. Saxitoxin in the Biomass

The wet biomass content of the polluted water (collected by filtration) was 30.4 g L^−1^, and dry biomass content was 3.13 g L^−1^. The dry biomass was subjected to analysis of saxitoxin by the method we published earlier [22] and it was found to be 5 ± 1 mg kg^−1^.

### 2.4. Saxitoxin in Water

**Figure 3:**
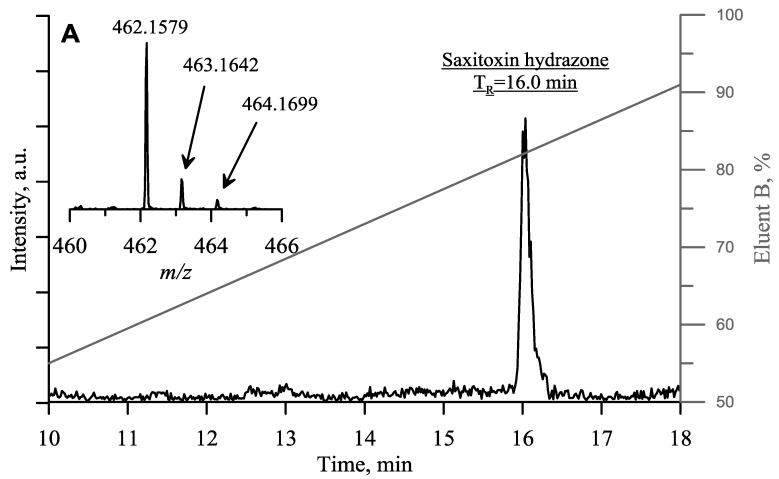
Extracted ion current chromatogram (*m*/*z* = 462.15 ± 0.10) of the water sample taken from the polluted area near the dam of the Irkutsk hydroelectric power station. Isotopic distribution of saxitoxin-2,4-dinitrophenylhydrazone is shown in the insert.

The method of analysis of saxitoxin in water based on HPLC-ESI-MS with pre-column modification of saxitoxin with 2,4-dinitrophenylhydrazine is described in the Section Materials and Methods. Figure 3 shows a chromatogram for the extracted positive ion with *m*/*z* 462.1579 corresponding to saxitoxin-2,4-dinitrophenylhydrozone. The retention time of the single peak obtained was the same as that of the saxitoxin-2,4-dinitrophenylhydrazone prepared from the saxitoxin standard. The insert in Figure 3 shows the isotopic ratio, which confirms that the identification was correct.

**Figure 4:**
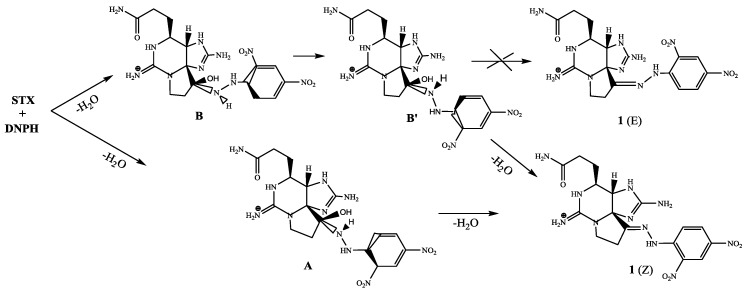
Mechanism of the reaction resulting in the *Z*-isomer of saxitoxin-2,4-dinitrophenyl hydrazone as suggested by quantum chemical calculations.

Many aldehydes and ketones can be detected by modification with 2,4-dinitrophenylhydrazine, although quantitative determination is complicated by formation of *E*- and *Z*-isomers. To overcome this issue, Uchiyama et al. suggested a protocol involving the reduction of formed hydrazone with NaBH_4_ or other reagents [23]. Our quantum chemical calculations suggested that in the case of saxitoxin only one isomer—the *Z*-isomer—can be formed due to strong steric hindrance preventing the formation of the *E*-isomer (Figure 4). This conclusion was in agreement with the fact that we found only one peak corresponding to saxitoxin-2,4-dinitrophenylhydrazone on the chromatogram shown in Figure 4.

The concentration of saxitoxin in the water from the polluted area was found to be 600 ± 100 μg L^−1^.

Immunoassay analysis was carried out by the Stylab Company (Moscow, Russia) according to the manufacturer’s instructions. The sample was diluted with deionized water to obtain a saxitoxin concentration appropriate for analysis. The concentration of saxitoxin was found to be 2900 ± 900 μg L^−1^. The discrepancy with HPLC-MS results can be explained by the fact that immunoassay analysis determines the total concentration of saxitoxin and all analogues present in the sample, while HPLC-MS specifically detects saxitoxin. Both methods revealed very high levels, with HPLC and immunoassay indicating concentrations 200 and ~1000 times higher (respectively) than the recommended health alert level for drinking water in Australia, Brazil and New Zealand, which is 3 μg L^−1^ [12]. The reported concentration of saxitoxin and its analogues in fresh water bodies varies over a wide range. For example, concentrations of 1.93 ± 0.64 μg L^−1^ of saxitoxin (measured by ELISA) have been reported for the coastal zone of Lake Baikal [10], while a particular site in a Finnish lake was found to contain 1070 μg L^−1^ saxitoxin [24]. Children who swam in the latter showed symptoms of poisoning, although these were different from those which would be caused by saxitoxin [24]. Saxitoxin-producing cyanobacteria and saxitoxin have been found in many fresh-water lakes, reservoirs and rivers [25,26,27,28].

### 2.5. Water Chemistry and Microbiology

The concentrations of nutrients in water from the polluted area were determined after filtration through a 0.45 μm filter, unless otherwise stated. Water was viscous, and rich foam appeared during filtration. The results are shown in Table 1 along with the data for the surface water of Lake Baikal for comparison [29,30,31,32]. The content of some nutrients such as ammonium, nitrate, inorganic phosphate, and total phosphorous were noticeably high in the polluted area compared to those in Lake Baikal (see Table 1). Suspended matter, permanganate and chemical oxygen demand (COD), potassium and bicarbonate concentrations were also significantly higher than those in Lake Baikal.

Microbiological analysis was performed to identify any traces of fresh fecal contamination. The total microbial count at 37 °C was found to be 10 CFU per 1 mL, total microbial count at 22 °C was 41 CFU per 1 mL, total coliforms were 2 CFU per 100 mL, thermotolerant coliforms were 2 CFU per 100 mL and no *Enterococci* and coliphages were detected. These results do not support the option of fecal pollution.

## 3. Discussion

We shall give below a summary of the new results and findings which are of potential interest to the scientific community that studies freshwater cyanotoxins in an effort to abate the risk of hazard to the users of toxin-containing fresh water.

*D. lemmermannii* had already been found in Lake Baikal and the Irkutsk Reservoir earlier [5,6,7,8,9,10,11]. It was identified as a dominating cyanobacteria in the plankton of Lake Baikal, sometimes producing large floating masses a few kilometers long [8,9]. In some years, *D. lemmermannii* has been found at very high concentrations—up to 10 × 10^6^ cells L^−1^ [9]. It was recently demonstrated that *D. lemmermannii* samples collected in and around Lake Baikal contain the *sxtA* gene involved in the synthesis of saxitoxins. STXs were detected by the immunoassay approach (ELISA) in *D. lemmermannii* in Baikal water within patches of the blooming cyanobacteria, although in small concentrations [10,11]. After we developed a new method of determination of STX based on precolumn derivatization with 2,4-dinitrophenylhydrazine followed by HPLC-ESI-MS, and for the first time applied it to analysis of polluted water as described in the present paper, we found that the concentration of STX in the patch of discolored water leaning against the dam of the Irkutsk hydroelectric power plant was extremely high, up to 600 μg L^−1^, a few hundred times higher than the health alert level (3 μg L^−1^) adopted for drinking water in some countries [12,15].

To find an explanation for this fact, we carried out hydrochemical and microbiological analyses of this STX-polluted water. The concentration of nutrients and some major ions appeared to be much higher than those usually found in surface water of the open Baikal and of the Irkutsk reservoir (see Table 1). However, microbiological studies did not confirm the possibility that the sources of nutrients could be a discharge of insufficiently treated municipal wastewater. Alternatively, we suggest that the high concentration of nutrients is due to secondary pollution, i.e., their delivery from degraded *D. lemmermannii* cells accumulated on the surface water near the dam due to the fact that the main stream of water goes to the turbines of the power station at a depth of 25 m. If our suggestion is correct, a similar situation could occur in other water reservoirs in Russia and worldwide. The authors hope that the data and techniques of analysis described in the present paper will attract the attention of authorities and decision-makers to the problem.

## 4. Materials and Methods

To study the morphology and fluorescence of constituents of water from the polluted area, light and epifluorescent microscopy was performed with an Axiovert 200 instrument (Carl Zeiss AG, Oberkochen, Germany) equipped with a mercury lamp (HBO 50) and four filters (UV, G 365, BP 450–490 and BP 546/12). The samples for the visualization of nucleic acids in akinetes were stained with 4′,6-diamidino-2-phenylindole (DAPI) solution [33]. Wet biomass was estimated by weighing the sample after centrifugation and removal of the supernatant, and dry biomass was estimated after complete desiccation of the wet biomass at 50 °C.

Isolation of DNA was carried out from a portion of the floating biomass collected from the surface of the polluted area. Phenol-chloroform extraction was used to isolate the total DNA from the floating biomass. The DNA was analyzed for the presence of the *sxtA* gene, which encodes a polyketide synthase that initiates synthesis of saxitoxin. Primers for *sxtA* were used to carry out a polymerase chain reaction (PCR). Amplification of the *sxtA* gene fragment has been used in published studies for the identification of various saxitoxin-producing cyanobacteria in fresh waters [21], including Lake Baikal [8,10]. Extracted DNA of the cyanobacteria *Aphanizomenon gracile* was taken as a positive control [11,16], and DNA of *Synechococcus* Bal 9710 was used as a negative control as this species does not contain the *sxtA* gene. The PCR, cloning and sequencing reactions were performed as described elsewhere [11,16]. Data were analyzed with the Mega 7.0 software package (Pennsylvania State University, State College, PA, USA, 2016) [34].

Analysis of saxitoxin in biomass was performed according to the method published earlier, which is based on HPLC-MS with precolumn modification [22]. Quantum chemical calculations were performed using Gaussian 09 software [35].

The saxitoxin in the polluted water was analysed using two methods: (i) HPLC-ESI-MS with pre-column modification of saxitoxin with 2,4-dinitrophenylhydrazine and (ii) ELISA immunoassay.

### 4.1. HPLC-MS

Detection of saxitoxin from the sampled water was carried out by first removing the solid phase by centrifugation (15 min, 10,000× *g*). Three 1 mL aliquots of the supernatant were put into separate 1.5 mL centrifuge tubes, then 1 and 2 μL of 10% (*v*/*v*) saxitoxin standard solution diluted in deionized water was added into tubes #2 and #3, respectively. We used the method of standard addition for calibration. The samples were dried by vacuum centrifugation at 60 °С, then 200 μL of 0.1% heptafluorobutyric acid (HFBA) in acetonitrile was added to the dry residue. Samples were sonicated for 30 min and centrifuged for 15 min at 10,000× *g*. Aliquots of the supernatant (160 μL) were put into glass vials, and the solvent removed by vacuum centrifugation at 60 °С. A solution of 10 mg mL^−1^ 2,4-dinitrophenylhydrazine in acetonitrile was supplemented with trifluoroacetic acid (98.5/1.5 *v*/*v*) and added to the dry residue. The vials were stoppered and incubated at 65 °С for 24 h. Deionized water (40 μL) and 150 μL of chloroform were added to each sample, and the tubes shaken for 5 min. Aliquots of the supernatants (30 μL) were analysed using an Agilent 1200 liquid chromatography system on a reversed phase Zorbax 300SB-C18 column (5 μm, 2.1 × 150 mm) coupled to an Agilent 6210 time-of-flight mass spectrometer with electrospray ionization. Eluent A was 0.1% HFBA in water, and eluent B was 0.1% HFBA in acetonitrile. The column was washed with 100% eluent B for 15 min at 0.2 mL min^−1^ followed by a mixture of eluent A (90%) and eluent B (10%) for 15 min at 0.2 mL min^−1^. Preconditioning of the column and the following chromatography was performed at 35° C. Elution was performed with a linear gradient of eluent (10% eluent B to 100% eluent B) at a flow rate of 0.15 mL min^−1^ for 20 min. The mass spectrometry detection mode was positive ion electrospray ionization (ESI+). The range of detection was 100–600 Da and the ion source temperature was set at 250° C. The chromatogram at *m*/*z* 462.15 ± 0.10 was extracted from the data set.

### 4.2. ELISA Immunoassay

The enzyme-linked immunosorbent assay (ELISA) of polluted water was performed using an Abraxis Saxitoxin ELISA kit (Abraxis LLC, Warminster, USA) according to the manufacturer’s protocol.

Determination of ammonia (NH_4_^+^), nitrite (NO_2_^−^) and phosphate (PO_4_^3−^) ions, as well as the total phosphorous (in filtered and unfiltered water), permanganate index, chemical oxygen demand (COD), pH and electric conductivity, were carried out according to the guidelines of the Russian Health Protection Agency [36]. Measurement of nitrate (NO_3_^−^) and bicarbonate (HCO_3_^−^) ions were carried out by HPLC [37,38]; sulfate (SO_4_^2−^) and chloride (Cl^−^) ions were analyzed by ion chromatography and sodium (Na^+^), potassium (K^+^), magnesium (Mg^2+^) and calcium (Ca^2+^) were analyzed by atomic absorption spectrometry. Analyses for these components were performed against water from the polluted area subjected to dialysis through a cellulose membrane (50 mL of polluted water vs. 350 mL of deionized water). Data obtained for dialysate were multiplied by eight.

Microbiological indices were determined according to the guidelines of the Russian Health Protection Agency–[39,40,41] under the following protocols:

*The total microbial count*. 1 mL of polluted water was inoculated to the Petri dishes with nutritive agar (peptone—10 g L^−1^, beef extract—500 g L^−1^, NaCl—5 g L^−1^, agar—25 g L^−1^, NICF, Saint Petersburg, Russia). This was done in triplicate, and then three Petri dishes were incubated at 37 °C for 24 h, and other three Petri dishes at 22 °C for 72 h. The plate counts of each dish were added together and divided by water volume in mL inoculated into the dishes.

*Total and thermotolerant coliforms.* Three portions of polluted water 100, 50 and 50 mL each were filtered through three membrane filters (0.45 μm pores, 47 mm in diameter). The filters were then put on Endo medium and incubated at 37 °C for 18-24 h. Dark red mucous colonies with metallic luster of lactose-positive bacteria were taken into account. After oxidase test, the oxidase-negative and gram-negative colonies were reinoculated in duplicate on Hiss medium with lactose. The first replicate was incubated at 37 °C for 48 h. The colonies were considered positive for the total coliforms when gas and acid were detected. The second replicate was incubated at 44 °C for 24 h. The colonies were considered positive for the thermotolerant coliforms when gas and acid were detected.

*Enterococci.* Three portions of polluted water 100, 50 and 50 mL each were filtered through three membrane filters (0.45 μm pores, 47 mm in diameter). The filters were then placed on Azide medium and incubated at 37 °C for 48 h. No *Enterococci* were detected.

*Coliphages*. 10 portions of water of 10 mL each were placed in 10 Petri dishes with a double concentrated nutritive agar. Water was sterilized with chloroform (1 mL of chloroform per 10 mL of water). The sample was thoroughly shaken and left for 15 min to separate chloroform. The supernatant was taken for further study. 1.0 mL of *E. coli* K_12_ F^+^ Str-r suspension was added to 100 mL of agar. 10 mL of water sample was placed into sterile Petri dishes and covered with 25 mL of nutritive agar with *E. coli*. The content of the dishes was stirred with care and left at room temperature to congeal. Then the dishes were turned upside down and incubated at 37 °C for 18 h. No plaque-forming units were found.

## Figures and Tables

**Table 1:** Results of hydrochemical analysis of water from the polluted area compared with the surface water of Lake Baikal.

Indicator	Polluted Area Near the Dam of the Irkutsk Reservoir, 1 September 2017	Surface Water of Southern Lake Baikal, Mean Values for the Last 5 Years
NH_4_^+^, mg L^−1^	10.8	0.0–0.01
NO_2_^−^, mg L^−1^	0.29	0.0–0.003
NO_3_^−^, mg L^−1^	9.72	0.1–0.26
PO_4_^3−^, mg L^−1^	6.8	0.003–0.01
Total P (filtered sample), mg L^−1^	2.5	0.001–0.01
Total P (unfiltered sample), mg L^−1^	11.0	No data
Permanganate index, mgО_2_ L^−1^	824	2.0
COD ^1^, mgО_2_ L^−1^	10,050	4.0
pH	6.13	8.08
E_c_^2^, μSm cm^−1^	646.70	109.9
Mineralisation	554.84	-
НСО_3_^−^, mg L^−1^	274.4	64.60
SO_4_^2−^, mg L^−1^	7.76	5.2
Cl^−^, mg L^−1^	1.6	0.45
Na^+^, mg L^−1^	12.0	3.36
K^+^, mg L^−1^	57.6	0.96
Ca^2+^, mg L^−1^	28.0	15.9
Mg^2+^, mg L^−1^	8.0	3.1

^1^ Chemical oxygen demand; ^2^ Electro conductivity.
